# Supplementary material for: Vaccine refusal in pregnant women in Kahramanmaraş: a community-based study from Türkiye
Source: PeerJ. 2024 May 20;12:e17409. doi: 10.7717/peerj.17409 (PMC11114108; doi:10.7717/peerj.17409)
Supplement: Supplemental Information 2 [file peerj-12-17409-s002.docx]

**SURVEY Family health center ( …………………. )**

1. Age: 2. Nationality: Turkish ……. Foreign……... Country…….

3. Week of pregnancy: 4. Which pregnancy is this:

5.How many children are there alive?......….

6. If so, how many ………

7. Have you vaccinated your living child(ren)?

a) Yes, all of it b) Yes, some of it c) No, I did not have it done

8. Any pregnancy-related disease ?............................................ .............

9. Place of residence: Dulkadiroğlu/ Onikişubat ?: Rural/Urban?:

10. Education Level: a ) Illiterate b)Literate c)Primary school d)Middle school e)High school f)University g)Master's degree

11. Educational status of spouse: a ) Illiterate b) Literate c)Primary school d)Middle school e)High school f)University g)Master's degree

12. Do you work? a) No b)Yes ............................................ ........................................................

13. Does your spouse work? a) No b)Yes ............................................ ........................................................

14. If any of them do work, are they in the healthcare sector? : a)Yes b)No

15. Income (Monthly-TL):

16. Will you have your baby vaccinated when she/he is born?

a) Yes, all of it b) Yes, but some of it c) No, I will not have it done at all.

17. (For pregnant women who chose option b in question 16) Which vaccinations do you plan not to have?

a) I don't know b) Write down the vaccine(s) that she will not have ............................................ .............

18. (For pregnant women who chose options b and c in question 16) Why won't you have it all done or not at all?

a) The idea that the vaccine will harm the baby's health

b) Do not think that the vaccine is useful/protective

c) The idea that the vaccine may cause infertility

d) The idea of not trusting vaccines in general

e) Religious beliefs/reasons

f) Recommendation of friends/relatives not to get vaccinated

g) Recommendation of healthcare personnel not to get vaccinated

h) Encountering someone who has experienced vaccine side effects

i) Other ........................................ ........................................................ .............

19. Can we learn your thoughts about the sentences given below?

1. If we do not vaccinate our children, diseases that we see very rarely today will re-emerge a ) True b)False

2. The whole society will benefit from vaccination a ) True b)False

3. Vaccination is unnecessary, if the baby is raised in a natural and healthy way, there is no need for vaccination a ) True b)False

4. The diseases that vaccines protect against are not that serious, so it is okay if they are not given. a ) True b)False

5. There may be some side effects immediately after vaccination a ) True b)False

6. Side effects of the vaccine appear after years a ) True b)False

7. Vaccines cause autism a ) True b)False

8. Vaccines weaken the immune system a ) True b)False

9. Since the baby is very small in the first 6 months, it would be better if the vaccinations were given later.

a) True b)False

10. Some vaccines are more dangerous than the diseases they protect against

a ) True b)False

11. Some vaccines contain heavy metals such as mercury

a ) True b)False

12. Healthcare professionals only talk about the benefits of the vaccine, not its harms a ) True b)False

13. I was not informed sufficiently by the healthcare professionals about the vaccines I will give my baby a ) True b)False

14. I encountered a baby/child/adult who was exposed to vaccine side effects----------

20. Sources of information about vaccination?

a) Friends/relatives

b) Healthcare personnel

c) Television d ) Internet

e) Newspaper-Book-magazine f) Other

21. How much do you trust the doctor about vaccines?

a) I don't trust at all b)I don't trust c)I trust d)I trust very much

22. Are routine vaccinations during infancy and childhood charged in Turkey?

a)Yes b)No

23. Do you know where routine vaccinations during infancy are administered in Turkey?

a)Yes ………………………….. b)No

24. Have you heard of herd immunity?

a)Yes b)No

**Thank you for participating in the survey.**
