# Supplementary material for: Vaccine refusal in pregnant women in Kahramanmaraş: a community-based study from Türkiye
Source: PeerJ. 2024 May 20;12:e17409. doi: 10.7717/peerj.17409 (PMC11114108; doi:10.7717/peerj.17409)
Supplement: Supplemental Information 3 [file peerj-12-17409-s003.docx]

**ANKET Aile sağlığı merkezi (………………….)**

1.Yaş: 2. Uyruk: Türk…….Yabancı....................Ülke…….

3. Gebelik haftası: 4. Kaçıncı gebelik:

5.Yaşayan çocuk var mı?....….

6. Varsa kaç tane………

7. Yaşayan çocuğunuza/çocuklarınıza aşılarını yaptırdınız mı?

a) Evet tamamını b) Evet bir kısmını c) Hayır yaptırmadım

8. Gebelikle ilgili herhangi bir hastalık?...........................................................

9. Yerleşim yeri: Dulkadiroğlu/Onikişubat?: Kır/Kent?:

10. Eğitim Durumu: a)OYD b)OY c)İlkokul d)Ortaokul e)Lise f)Üniversite g)Yüksek lisans

11. Eşinin eğitim durumu: a)OYD b)OY c)İlkokul d)Ortaokul e)Lise f)Üniversite g)Yüksek lisans

12. Çalışıyor mu? a) Hayır b)Evet...........................................................................................

13. Eşi çalışıyor mu? a) Hayır b)Evet...........................................................................................

14. Çalışıyorlarsa sağlık sektörü mü? : a)Evet b)Hayır

15. Gelir (Aylık-TL):

16. Bebeğiniz doğduğunda aşılarını yaptıracak mısınız?

a) Evet tamamını b) Evet ama bir kısmını c)Hayır hiç yaptırmayacağım

17. (16. Soruda b şıkkını seçen gebeler için) Hangi aşıları yaptırmamayı düşünüyorsunuz?

a) Bilmiyorum b)Yaptırmayacağı aşı/aşıları yazın......................................................

18. (16. Soruda b ve c şıklarını seçen gebeler için) Neden tamamını yaptırmayacaksınız/hiç yaptırmayacaksınız?

a) Aşının bebeğin sağlığına zarar vereceği düşüncesi

b) Aşının yararlı/koruyucu olmadığını düşünme

c) Aşının kısırlık yapabileceği düşüncesi

d) Aşılara genel olarak güvenmeme düşüncesi

e) Dini inanışlar/sebepler

f) Yakınlarının/akrabalarının aşı yaptırmama yönündeki tavsiyesi

g) Sağlık personelinin aşı yaptırmama yönündeki tavsiyesi

h) Aşı yan etkisi ile karşılaşmış birisi ile karşılaşma

ı) Diğer.................................................................................................................

19. Aşağıda verilen cümleler hakkında düşüncelerinizi öğrenebilir miyiz?

1. Eğer çocuklarımıza aşı yaptırmazsak bugün çok nadir gördüğümüz hastalıklar yeniden ortaya çıkar a) Doğru b)Yanlış

2. Aşılanmadan tüm toplum fayda görür a) Doğru b)Yanlış

3. Aşılanma gereksizdir, bebek doğal ve sağlıklı bir şekilde büyütülürse, aşıya gerek yoktur a) Doğru b)Yanlış

4. Aşıların koruduğu hastalıklar o kadar da ciddi hastalıklar değildir, o nedenle yapılmasa da olur a) Doğru b)Yanlış

5. Aşılamadan hemen sonra bir takım yan etkiler olabilir a) Doğru b)Yanlış

6. Aşının yan etkileri yıllar sonra ortaya çıkar a) Doğru b)Yanlış

7. Aşılar otizme neden olur a) Doğru b)Yanlış

8. Aşılar bağışıklık sistemini zayıflatır a) Doğru b)Yanlış

9. İlk 6 ayda bebek çok küçük olduğu için aşılar daha sonra yapılsa daha iyi olur

a) Doğru b)Yanlış

10. Bazı aşılar korudukları hastalıklardan daha tehlikelidir a) Doğru b)Yanlış

11. Bazı aşıların içinde civa gibi ağır metaller vardır a) Doğru b)Yanlış

12. Sağlık çalışanları aşının sadece faydalarından bahsediyor, zararlarından bahsetmiyor a) Doğru b)Yanlış

13. Bebeğime yaptıracağım aşılar konusunda sağlık çalışanları tarafından yeterince bilgilendirilmedim a) Doğru b)Yanlış

14. Aşı yan etkisine maruz kalmış bir bebek/çocuk/yetişkin ile karşılaştım----------

20.Aşı hakkında bilgi kaynakları?

a) Arkadaşlar/yakınlar/akrabalar

b) Sağlık personeli

c) Televizyon d) İnternet

e)Gazete- Kitap-dergi f) Diğer

21. Aşılar hakkında hekime ne kadar güveniyorsunuz?

a) Hiç güvenmiyorum b)Güvenmiyorum c)Güveniyorum d)Çok güveniyorum

22. Türkiye’de bebeklik ve çocukluk döneminde rutin olarak yapılan aşılar ücretli midir?

a)Evet b)Hayır

23. Türkiye’de bebeklik döneminde rutin olarak yapılan aşıların nerede yapıldığını biliyor musunuz?

a)Evet ………………………….. b)Hayır

24. Toplum bağışıklığı/sürü bağışıklığı (toplumsal bağışıklık) diye bir şey duydunuz mu?

a)Evet b)Hayır

**Ankete Katıldığınız için Teşekkür ederiz.**
